# Supplementary material for: The First Use of the Washington Group Short Set in a National Survey of Japan: Characteristics of the New Disability Measure in Comparison to an Existing Disability Measure
Source: Int J Environ Res Public Health. 2024 Dec 10;21(12):1643. doi: 10.3390/ijerph21121643 (PMC11675656; doi:10.3390/ijerph21121643)
Supplement: Supplementary file 1 [file ijerph-21-01643-s001.zip › Table S1.pdf]

Table S1. Relevant variables for current study analysis

【The Washington Group Shor Set】

Questions

1. *Do you have difficulty seeing, even if wearing glasses?*
2. *Do you have difficulty hearing, even if using a hearing aid?*
3. *Do you have difficulty walking or climbing steps?*
4. *Do you have difficulty remembering or concentrating?*
5. *Do you have difficulty (with self-care such as) washing all over or dressing?*
6. *Using your usual (customary) language, do you have difficulty communicating, for example understanding or being understood?*

Answer options

1. *No - no difficulty*
2. *Yes – some difficulty*
3. *Yes – a lot of difficulty*
4. *Cannot do at all*

Categorization for analysis

1. Disability

Respondents who answered "a lot of difficulty" or "can not to do at all" to any of the six question

2. No disability

All answer patterns other than above mentioned "Disability"

【Existing measure on disability】

Questions (Original Japanese)

あなたは現在、健康上の問題で日常生活に何か影響がありますか。

Questions (English, translated by authors)

Does your health condition affect your daily life?

Answer options（Original Japanese）

1. *Yes (ある)*
2. *No (ない)*

Categorization for analysis

1. Disability (Yes)
2. No disability (No)

【Demographic variables】

<u>Sex</u>

Questions

Not applicable.

Answer options

1. *Male*
2. *Female*

Categorization for analysis

1. Male
2. Female

<u>Age</u>

Questions

Not applicable.

Answer options

Filling in birth of month, year.

Categorization for analysis

1. 6 to 19 years old, child
2. 20 to 39 years old, adult
3. 40 to 59 years old, middle
4. 60 to 79 years old, older
5. 80 and older, oldest-old

<u>Marital status</u>

Questions

Not applicable.

Answer options

1. *Married*
2. *Single*
3. *Widowed*
4. *Divorced*

Categorization for analysis

1. Married
2. Single
3. Divorced/widowed

Living area

Questions

Not applicable.

Answer options

1. Area with 500,000 or more inhabitants

2. 150,000 ≦ inhabitants < 500,000

3. 5,000 ≦ inhabitants < 150,000

4. inhabitants < 50,000

5. Other areas (in Japanese being described as “Gunbu”)

Categorization for analysis

1. Areas with 150,000 or more inhabitants
2. Areas with less than 150,000 inhabitants and other areas

【Health conditions】
Questions (Original, Japanese)
質問 1.
あなたは現在、傷病（病気やけが）で病院や診療所（医院、歯科医院）、 あんま・はり・きゅう・柔道整復師（施術所）に通っていますか?

補問. （質問 1 で「通っている」と回答した場合）
どのような傷病（病気やけが）で通っていますか。あてはまる全ての傷病名の番号に〇をつけてください。その中で最も気になる傷病名の番号を番号記入欄に記入してください。

Questions (English, translated by authors)
Question1.
Do you, currently, have a constant visit to hospital, clinic, acupuncture, moxibustion, Japanese massage, or Judo therapy for your health conditions (diseases or injuries)?

Question2. (Only respondents who choose answer option "Yes I do" in the qustion1)
What kinds of diseases or health conditions necessitate you to have the constant visit to hospital or others. Select answers that describe your conditions (multiple choice). Moreover, select one condition that is considered as the most concern condition for you.

Answer options（Original Japanese）
Question1.
1. *Yes (通っている)*
2. *No (通っていない)*

Question2.
1. *Diabetes (糖尿病)*
2. *Obesity (肥満症)*
3. *dyslipidemia (脂質異常症)*
4. *Diseases of thyroid (甲状腺の病気)*
5. *Depression and other mental conditions (うつ病やその他のこころの病気)*
6. *Dementia (認知症)*
7. *Parkinson's disease (パーキンソン病)*
8. *Other neurological diseases (その他の神経の病気)*
9. *Eye diseases (目の病気)*
10. *Ear diseases (耳の病気)*

11. Hypertension (高血圧症)
12. Stroke (脳卒中)
13. Angina pectoris/Myocardial infarction (狭心症・心筋梗塞)
14. Other circulatory diseases (その他の循環器系の病気)
15. Acute pharyngitis (急性鼻咽頭炎)
16. Allergic nasal inflammation　(アレルギー性鼻炎)
17. Chronic obstructive pulmonary disease (慢性閉塞性肺疾患)
18. asthma (喘息)
19. Other respiratory diseases　(その他の呼吸器系の病気)
20. Stomach/duodenum diseases(胃・十二指腸の病気)
21. Liver/gallbladder disease (肝臓・胆のうの病気)
22. Other digestive diseases (そのほかの消化器系の病気)
23. Dental diseases (歯の病気)
24. Atopic skin disease (アトピー性皮膚疾患)
25. Other skin disease (その他の皮膚の病気)
26. Gout (痛風)
27. Rheumatoid arthritis (関節リウマチ)
28. Arthritis (関節症)
29. Stiff shoulders (肩こり症)
30. Low back pain (腰痛症)
31. Osteoporosis (骨粗鬆症)
32. Kidney diseases (腎臓の病気)
33. Benign prostatic hyperplasia (前立腺肥大症)
34. Menopausal or postmenopausal disorders (閉経期又は閉経後障害)
35. Fracture(骨折)
36. Injury or burn other than fracture (骨折以外のけが・やけど)
37. Anemia/blood disease (貧血・血液の病気)
38. Malignant neoplasm (悪性新生物)
39. Pregnancy/puerperium (妊娠・産褥)
40. Infertility (不妊症)
41. Others (その他)
42. Unknown (不明)

【Physiological and psychosocial variables】

Subjective health status

Questions (Original Japanese)

あなたの現在の健康状態はいかがですか?

Questions (English, translated by authors)

What do you think of your general health condition?

Answer options（Original Japanese)

1. *Very good (よい)*
2. *Good (まあよい)*
3. *Normal(ふつう)*
4. *Bad (あまりうよくない)*
5. *Very bad (よくない)*

Categorization for analysis

1. *Very good / good*
2. *Normal*
3. *Very bad / bad*

Ethyl alcohol consumptions

Questions (Original Japanese)

質問 1

あなたは週に何日くらいお酒(清酒、焼酎、ビール、洋酒など)を飲みますか?

補問

お酒を飲む日は 1 日あたり、どのくらいの量を飲みますか。

Questions (English, translated by authors)

Quesiton1

How often do you drink alcohol?

Quesiton2

How much alcohol do you drink in a day?

Answer options（Original Japanese)

Quesiton1

1. *Every day (毎日)*
2. *Five to six days per week (週 5-6 日)*
3. *Three to four days per week (週 3-4 日)*
4. *One to two days per week (週 1-2 日)*
5. *One to three days per a month (月 1-3 日)*
6. *I seldom drink alcohol (ほとんど飲まない)*
7. *I quit drinking alcohol (やめた)*

Quesiton2

1. *Less than 180 ml (1 合（180ml）未満)*
2. *180ml ≦ alcohol drink < 360ml (1 合以上 2 合（360ml）未満)*
3. *360ml ≦ alcohol drink < 540ml (2 合以上 3 合（540ml）未満)*
4. *540ml ≦ alcohol drink < 720ml (3 合以上 4 合（720ml）未満)*
5. *720ml ≦ alcohol drink < 900ml (4 合以上 5 合（900ml）未満)*
6. *900ml ≦ alcohol drink (5 合（900ml）以上)*

Categorization for analysis †

1. Never or quit drink
2. Social drinker or low risk group (> 0 to ≤ 100 g/week)
3. Middle risk group (> 100 to ≤ 350 g/week)
4. High risk group (>350 g/week)

†

Following a previous study(††), we equivalized 180ml alcohol drink with 20g ethyl alcohol for estimating the amount of ethyl alcohol consumptions (g) per week. Furthermore, we interpreted each answer option as followed:

Question1: *every day*; 7 days/week, *five to six days per week*; 6 days/week, *three to four days per week*; 4 days/week; *one to two days per week*; 2 days/week; *one to three days per a month*; 0.7 days/week and, *I seldom drink alcohols*, or *I quit drinking alcohols* or *I do not drink alcohols*; 0 day/week

Question2: *less than 180 ml*; 90 ml/day, *180ml ≦ alcohols < 360ml*; 270ml/day, *360ml ≦ alcohols < 540ml*; 450ml/day, *540ml ≦ alcohols < 720ml*; 630ml/day, *720ml ≦ alcohols < 900ml*; 810ml/day and, *900ml ≦ alcohols*; 990ml/day

The weekly ethyl alcohol consumption was estimated by multiplying the frequency (answer of question1) and amount of alcohol consumption (answer of question2).

††Kawamura C, Iwagami M, Sun Y, Komiyama J, Ito T, Sugiyama T, Bando H, Tamiya N: **Factors associated with non-participation in breast cancer screening: analysis of the 2016 and 2019 comprehensive survey of living conditions in Japan**. *Breast Cancer* 2023, **30**(6):952-964.

Smoking habit

Questions (Original Japanese)

あなたはたばこを吸いますか？

Questions (English, translated by authors)

Do you smoke?

Answer options（Original Japanese)

1. *Every day (毎日吸っている)*
2. *Sometimes (時々吸う日がある)*
3. *I quit smoking for more than a month (以前は吸っていたが1か月以上吸っていない)*
4. *I do not smoke (吸わない)*

Categorization for analysis

1. Never/ex-smoker (*I quit smoking for more than a month / I do not smoke*)
2. Current smoker (*Every day / Sometimes*)

Educational qualification

Questions

Not applicable.

Answer options

1. *Primary and junior high school (小学・中学)*
2. *High school (高校・旧性中)*
3. *Vocational school　(専門学校)*
4. *Community college or technical college (短大・高専)*
5. *University (大学)*
6. *Post graduate school (大学院)*

Categorization for analysis

1. Vocational school/community(technical) college/university/post graduate school
2. High school

3. Primary/junior high school

Subjective financial state

Questions (Original Japanese)

現在の暮らしの状況を総合的にみて、どう感じていますか。

Questions (English, translated by authors)

What do you think your current general economic situation?

Answer options（Original Japanese)

1. *Very poor (大変苦しい)*
2. *Poor (やや苦しい)*
3. *Normal (普通)*
4. *Wealthy (ややゆとりがある)*
5. *Very wealthy (大変ゆとりがある)*

Categorization for analysis

1. Wealthy (Very wealthy / wealthy)
2. Normal (Normal)
3. Poor (Very poor / poor)

Kessler Psychological Distress Scale

Questions (Original Japanese)

次の(1)から(6)の質問について、過去 1 か月の間どうでしたか？

1. *神経過敏に感じましたか*
2. *絶望的だと感じましたか*
3. *そわそわ、落ち着かなく感じましたか*
4. *気分が沈み込んで、何が起こっても気が晴れないように感じましたか*
5. *何をするのも骨折りだと感じましたか*
6. *自分は価値のない人間だと感じましたか*

Questions (English, translated by authors)

The following questions are about how you have been feeling during the past 30 days.

1. *How often did you feel nervous?*
2. *How often did you feel hopeless?*

3. *How often did you feel restless or fidgety?*
4. *How often did you feel so depressed that nothing could cheer you up?*
5. *How often did you feel everything was an effort?*
6. *How often did you feel worthless?*

Answer options（Original Japanese）

1. All (いつも)
2. Most （たいてい)
3. Some (ときどき)
4. A little (少しだけ)
5. None (まったくない)

Categorization for analysis †

1. Normal (total score =< 4)
2. Mild illness (5 ≦ total score ≦ 12)
3. Severe illness (13 ≦ total score)

† Kawamura C, Iwagami M, Sun Y, Komiyama J, Ito T, Sugiyama T, Bando H, Tamiya N: **Factors associated with non-participation in breast cancer screening: analysis of the 2016 and 2019 comprehensive survey of living conditions in Japan**. *Breast Cancer* 2023, **30**(6):952-964.

<u>Health insurance</u>

Questions

Not applicable.

Answer options

1. *Being insured for National Health Insurance (国民健康保険)*
2. *Being insured for Employee insurance (被用者保険)*
3. *Being insured for Medical care insurance for older adults aged 75 years and over (後期高齢者医療制度)*
4. *Being insured for Other (その他)*

Categorization for analysis

1. National Health Insurance
2. Employee insurance
3. Other (*Medical care insurance for older adults aged 75 years and over or other*)

Employment status

Questions (Original Japanese)

質問 1

仕事の状況

補問

勤めか自営かの別（質問 1 で「仕事あり」と回答した場合）

Questions (English, translated by authors)

Quesiton1

Working situation

Quesiton2 (Only respondents who choose answer option “1” in the qustion1)

Types of work

Answer options（Original Japanese）

Quesiton1

1. Working (mainly working, housework, schooling or others) (仕事あり（主に仕事をしている、主に家事で仕事あり、主に通学で仕事あり、その他））

2. Not-working (housework, schooling or others) (仕事なし（通学、家事、その他））

Quesiton2

1. Employee (contract period: permanent) (一般常勤者（契約期間の定めのない雇用者））
2. Employee (contract period: more than a month) (一般常勤者（契約期間が 1 か月以上の雇用者））
3. Contract employee(1 month ≦contract period ≦ 1 year) (1 月以上 1 年未満の契約の雇用者)
4. Contract employee (contract period < 1 month) (日々又は 1 月未満の契約の雇用者)
5. Executive of company or corporation (会社・団体等の役員)
6. Self-employed (with employee) (自営業者（雇人あり））
7. Self-employed (without employee) (自営業者（雇人なし））
8. Family worker (家族従業者（自家営業の手伝い））
9. Doing piecework at home (内職)
10. Other (その他)

Categorization for analysis †

1. Employed

2. Self-employed

3. Employed(other)

4. Unemployed

†

We considered employment status as:

Unemployed; Answer of Quesiton1, Not-working

Employed; “Answer of Quesiton1, Working” AND “Answer of Quesiton2, 1–4”

Self- employed; Answer of Quesiton1, AND “Answer of Quesiton2, 6–7”

Employed(others); Answer of Quesiton1, AND “Answer of Quesiton2, 5 or 8–10”

End
