# Supplementary material for: The First Use of the Washington Group Short Set in a National Survey of Japan: Characteristics of the New Disability Measure in Comparison to an Existing Disability Measure
Source: Int J Environ Res Public Health. 2024 Dec 10;21(12):1643. doi: 10.3390/ijerph21121643 (PMC11675656; doi:10.3390/ijerph21121643)
Supplement: Supplementary file 1 [file ijerph-21-01643-s001.zip › Table S2.pdf]

Table S2. Number and percentage of data with missing value. These data were excluded from the final analysis.(n=41,540)

	Number	(Percent, %)
Sex	0	(0)
Age	0	(0)
Marital status	0	(0)
Educational qualification	5116	(12.32)
Living area	0	(0.00)
Subjective health status	435	(1.05)
Ethyl alcohol consumptions	544	(1.31)
Smoking habit	473	(1.14)
Subjective financial state	0	(0.00)
Kessler Psychological Distress Scale	1579	(3.80)
Health insurance	253	(0.61)
Employment status	1102	(2.65)
Constant visit to hospitals	218	(0.52)
The most concerned health condition	1447	(3.48)
Disability defined by existing measure of disability	794	(1.91)
WGSS, vision	850	(2.05)
WGSS, hearing	2288	(5.51)
WGSS, mobility	797	(1.92)
WGSS, cognition	927	(2.23)
WGSS, self-care	849	(2.04)
WGSS, communication	809	(1.95)

NOTES: WGSS; Washington Group Short Set on Functioning
