# Supplementary material for: The First Use of the Washington Group Short Set in a National Survey of Japan: Characteristics of the New Disability Measure in Comparison to an Existing Disability Measure
Source: Int J Environ Res Public Health. 2024 Dec 10;21(12):1643. doi: 10.3390/ijerph21121643 (PMC11675656; doi:10.3390/ijerph21121643)
Supplement: Supplementary file 1 [file ijerph-21-01643-s001.zip › Table S3.pdf]

Table S3. Chi square test and binomial logistic regression analysis for the disagreement between the two disability measures that relatively lowered the disability prevalence estimates defined by the WGSS compared with that defined by the existing disability measure (participants aged 20 years and over)

	Disability				Chi square test	Binomial logistic regression [d)]	
	Existing measure; No WGSS; No (n=22551)		Existing measure; Yes WGSS: No (n=2232)				
	Number	(Percent)	Number	(Percent)	P-value	Adjusted Odds ratio	(95% confidence interval)
Sex					0.001		
Male	10894 †	(48.31)	999	(44.76)		Reference	
Female	11657 †	(51.69)	1233	(55.24)		0.90	(0.80 – 1.01)
Age (years)					<0.001		
20–39	4979 †	(22.08)	244	(10.93)		Reference	
40–59	8155 †	(36.16)	578	(25.90)		1.23	1.01 – 1.48
60–79	8060 †	(35.74)	1104	(49.46)		1.51	1.21 – 1.89
80 and over	1357 †	(6.02)	306	(13.71)		1.80	1.32 – 2.44
Marital status					<0.001		
Married	15061 †	(66.79)	1404	(62.90)		Reference	
Single	4862 †	(21.56)	389	(17.43)		1.13	(0.96 – 1.32)
Divorced/widowed	2628 †	(11.65)	439	(19.67)		1.27	(1.09 – 1.47)
Living area					0.97		
Area with 150,000 or more inhabitants	10812	(47.94)	1071	(47.98)		Reference	
Area with less than 150,000 inhabitants	11739	(52.06)	1161	(52.02)		1.03	(0.93 – 1.14)
The most concerned health condition [a)]; low back pain					<0.001		
No [b)]	22102 †	(98.01)	2022	(90.59)		Reference	
Yes [c)]	449 †	(1.99)	210	(9.41)		1.76	(1.42 – 2.17)
The most concerned condition [a)]; depression and other mental conditions					<0.001		

No [b]	22316	†	(	98.96	)	2059	(	92.25	)		Reference					
Yes [c]	235	†	(	1.04	)	173	(	7.75	)		1.66	(	1.28	–	2.15	)
The most concerned health condition [a]; hypertension										<0.001						
No [b]	20286	†	(	89.96	)	2087	(	93.50	)		Reference					
Yes [c]	2265	†	(	10.04	)	145	(	6.50	)		0.33	(	0.27	–	0.40	)
The most concerned health condition [a]; diabetes										0.003						
No [b]	21624	†	(	95.89	)	2111	(	94.58	)		Reference					
Yes [c]	927	†	(	4.11	)	121	(	5.42	)		0.56	(	0.44	–	0.70	)
The most concerned health condition [a]; arthritis										<0.001						
No [b]	22363	†	(	99.17	)	2116	(	94.80	)		Reference					
Yes [c]	188	†	(	0.83	)	116	(	5.20	)		2.68	(	2.02	–	3.55	)
The most concerned health condition [a]; dyslipidemia										0.002						
No [b]	21852	†	(	96.90	)	2189	(	98.07	)		Reference					
Yes [c]	699	†	(	3.10	)	43	(	1.93	)		0.38	(	0.27	–	0.53	)
The most concerned health condition [a]; dental diseases										0.02						
No [b]	21923	†	(	97.22	)	2188	(	98.03	)		Reference					
Yes [c]	628	†	(	2.78	)	44	(	1.97	)		0.43	(	0.30	–	0.60	)
The most concerned health condition [a]; eye diseases										0.39						
No [b]	22030		(	97.69	)	2174	(	97.40	)		Reference					
Yes [c]	521		(	2.31	)	58	(	2.60	)		0.47	(	0.34	–	0.64	)
Subjective health status										<0.001						
Good	9691	†	(	42.97	)	228	(	10.22	)		Reference					
Normal	11761	†	(	52.15	)	896	(	40.14	)		2.24	(	1.92	–	2.61	)
Bad	1099	†	(	4.87	)	1108	(	49.64	)		18.1	(	15.3	–	21.5	)
Ethyl alcohol consumptions										<0.001						
Never or quit drink	12697	†	(	56.30	)	1457	(	65.28	)		Reference					
Social drinker or low risk group (> 0 to ≤ 100 g/week)	5157	†	(	22.87	)	419	(	18.77	)		0.87	(	0.76	–	1.00	)
Middle risk group (> 100 to ≤ 350 g/week)	4001	†	(	17.74	)	305	(	13.66	)		0.87	(	0.74	–	1.03	)

High risk group (> 350 g/week)	696 †	(3.09)	51	(2.28)		0.87	(0.61 – 1.24)
Smoking habit					<0.001		
Never/ex-smoker	18874 †	(83.69)	1961	(87.86)		Reference	
Current smoker	3677 †	(16.31)	271	(12.14)		0.92	(0.78 – 1.08)
Educational qualification					<0.001		
Primary/junior high school	1926 †	(8.54)	287	(12.86)		Reference	
High school	9502 †	(42.14)	994	(44.53)		1.14	(0.96 – 1.36)
Vocational school/junior college/community(technical) college/university/post graduate school	11123 †	(49.32)	951	(42.61)		1.42	(1.18 – 1.72)
Subjective financial state					<0.001		
Wealthy	1429	(6.34)	131	(5.87)		Reference	
Nor poor not wealthy	9789 †	(43.41)	837	(37.50)		0.76	(0.61 – 0.96)
Poor	11333 †	(50.25)	1264	(56.63)		0.88	(0.71 – 1.10)
Kessler Psychological Distress Scale					<0.001		
Normal (total score =< 4)	17987 †	(79.76)	1259	(56.41)		Reference	
Mild illness (5 ≦ total score ≦ 12)	4058 †	(17.99)	755	(33.83)		1.61	(1.42 – 1.82)
Severe illness (13 ≦ total score)	506 †	(2.24)	218	(9.77)		2.84	(2.25 – 3.59)
Health insurance					<0.001		
Employee insurance	13956 †	(61.89)	860	(38.53)		Reference	
National Health Insurance	5598 †	(24.82)	748	(33.51)		1.21	(1.03 – 1.42)
Other	2997 †	(13.29)	624	(27.96)		1.37	(1.11 – 1.69)
Employment status					<0.001		
Employed	12739 †	(56.49)	736	(32.97)		Reference	
Self-employed	1499	(6.65)	150	(6.72)		1.38	(1.08 – 1.75)
Employed(other)	1503	(6.66)	137	(6.14)		1.37	(1.09 – 1.73)
Unemployed	6810	(30.20)	1209	(54.17)		1.70	(1.45 – 1.99)

a) The condition which necessitates a study participant to visit hospital constantly and is also considered as the most concerned condition among his/her conditions.

b) “No” includes study participants who do not visit hospital constantly or who visit hospital constantly but do not have the specific condition nor consider the specific condition as the most concern.

c) “Yes” includes study participants who visit hospital constantly due to the specific condition that is considered as the most concern condition. The health conditions that are considered as the most concerning condition among at least 5% of study participants who constantly visit hospital in either group (Existing measure-No and WGSS-No (n=9879), Existing measure-Yes and WGSS-No (n=1908)) are only incorporated in the multivariate logistic regression analysis.

d) All variables were incorporated in the analysis model using forced entry method

† The adjusted residual values exceeded 1.96 or below -1.96
