# Supplementary material for: The First Use of the Washington Group Short Set in a National Survey of Japan: Characteristics of the New Disability Measure in Comparison to an Existing Disability Measure
Source: Int J Environ Res Public Health. 2024 Dec 10;21(12):1643. doi: 10.3390/ijerph21121643 (PMC11675656; doi:10.3390/ijerph21121643)
Supplement: Supplementary file 1 [file ijerph-21-01643-s001.zip › Table S4.pdf]

Table S4. Chis square test and binomial logistic regression analysis for the disagreement between the two disability measures that relatively increased the disability prevalence estimates defined by the WGSS compared with that defined by the existing disability measure (participants aged 20 years and over)

	Disability														
	WGSS; Yes Existing measure; Yes (n=1800)				WGSS; Yes Existing measure; No (n=1424)				Chi square test	Binomial logistic regression [d]					
	Number	(	Percent	)	Number	(	Percent	)	P-value	Adjusted Odds ratio	(	95% confidence interval			)
Sex									<0.001						
Male	697 †	(	38.72	)	680	(	47.75	)		Reference					
Female	1103 †	(	61.28	)	744	(	52.25	)		0.96	(	0.77	–	1.18	)
Age									<0.001						
80 and over	885 †	(	49.17	)	360	(	25.28	)		Reference					
60–79	612	(	34.00	)	461	(	32.37	)		1.42		1.04		1.92	
40–59	205 †	(	11.39	)	372	(	26.12	)		1.87		1.19		2.93	
20–39	98 †	(	5.44	)	231	(	16.22	)		2.79		1.60		4.84	
Marital status									<0.001						
Married	943 †	(	52.39	)	824	(	57.87	)		Reference					
Single	189 †	(	10.50	)	250	(	17.56	)		0.71	(	0.50	–	0.99	)
Divorced/widowed	668 †	(	37.11	)	350	(	24.58	)		0.95	(	0.75	–	1.20	)
Living area									0.88						
Area with 150,000 or more inhabitants	817	(	45.39	)	650	(	45.65	)		Reference					
Area with less than 150,000 inhabitants	983	(	54.61	)	774	(	54.35	)		1.16	(	0.97	–	1.39	)
The most concerned health condition [a]; hyperextension									0.01						
No [b]	1615 †	(	89.72	)	1236	(	86.80	)		Reference					
Yes [c]	185 †	(	10.28	)	188	(	13.20	)		1.63	(	1.23	–	2.17	)
The most concerned health condition [a]; low back pain									<0.001						

No [b)]	1647 †	(91.50)	1366	(95.93)		Reference	(	–)
Yes [c)]	153 †	(8.50)	58	(4.07)		0.90	(0.62	– 1.32)
The most concerned health condition [a)]; depression and other mental conditions					<0.001			
No [b)]	1690 †	(93.89)	1400	(98.31)		Reference		
Yes [c)]	110 †	(6.11)	24	(1.69)		0.46	(0.27	– 0.81)
The most concerned health condition [a)]; dementia					<0.001			
No [b)]	1696 †	(94.22)	1406	(98.74)		Reference		
Yes [c)]	104 †	(5.78)	18	(1.26)		0.36	(0.21	– 0.64)
The most concerned health condition [a)]; arthritis					<0.001			
No [b)]	1697 †	(94.28)	1401	(98.38)		Reference		
Yes [c)]	103 †	(5.72)	23	(1.62)		0.46	(0.28	– 0.78)
The most concerned health condition [a)]; eye diseases					0.02			
No [b)]	1703 †	(94.61)	1373	(96.42)		Reference		
Yes [c)]	97 †	(5.39)	51	(3.58)		1.03	(0.67	– 1.57)
The most concerned health condition [a)]; diabetes					0.06			
No [b)]	1709	(94.94)	1330	(93.40)		Reference		
Yes [c)]	91	(5.06)	94	(6.60)		2.25	(1.56	– 3.25)
Subjective health status					<0.001			
Bad	1220 †	(67.78)	250	(17.56)		Reference		
Normal	488 †	(27.11)	789	(55.41)		6.00	(4.90	– 7.35)
Good	92 †	(5.11)	385	(27.04)		11.0	(8.12	– 14.9)
Ethyl alcohol consumptions					<0.001			
Never or quit drink	1393 †	(77.39)	921	(64.68)		Reference		
Social drinker or low risk group (> 0 to ≤ 100 g/week)	244 †	(13.56)	241	(16.92)		1.03	(0.80	– 1.34)
Middle risk group (> 100 to ≤ 350 g/week)	143 †	(7.94)	219	(15.38)		1.21	(0.88	– 1.66)
High risk group (> 350 g/week)	20 †	(1.11)	43	(3.02)		2.37	(1.16	– 4.85)
Smoking habit					<0.001			

Never/ex-smoker	1651 †	(91.72)	1221	(85.74)		Reference	
Current smoker	149 †	(8.28)	203	(14.26)		1.05	(0.77 – 1.44)
Educational qualification					<0.001		
Vocational school/junior college/community(technical) college/university/post graduate school	408 †	(22.67)	479	(33.64)		Reference	
High school	776	(43.11)	625	(43.89)		1.07	(0.85 – 1.35)
Primary/junior high school	616 †	(34.22)	320	(22.47)		1.13	(0.86 – 1.50)
Subjective financial state					0.26		
Wealthy	79	(4.39)	80	(5.62)		Reference	
Nor poor not wealthy	679	(37.72)	538	(37.78)		0.94	(0.60 – 1.46)
Poor	1042	(57.89)	806	(56.60)		0.97	(0.63 – 1.50)
Kessler Psychological Distress Scale					<0.001		
Severe illness (13 ≦ total score)	269 †	(14.94)	103	(7.23)		Reference	
Mild illness (5 ≦ total score ≦ 12)	764 †	(42.44)	403	(28.30)		1.26	(0.90 – 1.77)
Normal (total score =< 4)	767 †	(42.61)	918	(64.47)		1.89	(1.34 – 2.67)
Health insurance					<0.001		
Other	1137 †	(63.17)	495	(34.76)		Reference	
Employee insurance	300 †	(16.67)	631	(44.31)		1.22	(0.82 – 1.82)
National Health Insurance	363	(20.17)	298	(20.93)		1.05	(0.76 – 1.46)
Employment status					<0.001		
Unemployed	1444 †	(80.22)	674	(47.33)		Reference	
Employed	201 †	(11.17)	587	(41.22)		2.53	(1.82 – 3.51)
Self-employed	79 †	(4.39)	87	(6.11)		1.68	(1.12 – 2.53)
Employed(other)	76	(4.22)	76	(5.34)		1.09	(0.71 – 1.68)

a) The condition which necessitates a study participant to have constant hospital visit and is also considered as the most concerned condition among his/her conditions

b) "No" includes study participants who do not visit hospital constantly or who visit hospital constantly but do not have the specific condition nor consider the specific condition as the most concern.

c) “Yes” includes study participants who visit hospital constantly due to the specific condition that is considered as the most concern condition. The health conditions that were considered as the most concerning condition among at least 5% of study participants who visit hospital constantly in either group (Existing measure-Yes and WGSS-Yes (n=1645); Existing measure-No and WGSS-Yes (n=869)) are only incorporated in the multivariate logistic regression analysis.

d) All variables were incorporated in the analysis model using forced entry method.

† The adjusted residual values exceeded 1.96 or below -1.96
